# Objective risk assessment of bark and ambrosia beetles non‐indigenous to North America

**DOI:** 10.1002/eap.70072

**Published:** 2025-07-09

**Authors:** Andrew J. Johnson, David Bednar, Jiri Hulcr

**Affiliations:** ^1^ School of Forest, Fisheries and Geomatics Sciences, University of Florida Gainesville Florida USA; ^2^ Florida State Collection of Arthropods, FDACS‐DPI Gainesville Florida USA; ^3^ USDA‐APHIS‐PPQ, Science & Technology, Plant Pest Risk Analysis Raleigh North Carolina USA

**Keywords:** adventive, exotic, invasive, pest categorization, risk analysis, Scolytinae, woodborers

## Abstract

Pest risk assessment informs regulatory decisions to facilitate safe trade while also protecting a country's agricultural and environmental resources. The first step in pest risk assessment is pest categorization which can help determine whether an in‐depth examination is needed. We created a model to predict the potential impact of non‐indigenous bark and ambrosia beetles (Curculionidae: Scolytinae). This model uses biological variables derived from extensive assessment of alien species and produces a five‐point scale of impact prediction. We accommodate uncertainty and missing data using random decision tree forests with Monte Carlo simulations. Non‐indigenous bark beetles include both invasive species with significant ecological impacts, such as widespread tree death, and others that pose little risk. We assembled a comprehensive list of 60 introduced non‐native bark beetle species in the continental United States as the training set. Forty‐two potentially predictive variables were chosen from reports on behaviors, pestilence, recorded damage/interpretations in literature, biological traits, and interactions with fungi including plant pathogens. The model builds upon strategies used by USDA‐APHIS in existing risk assessments, specifically the Objective Prioritization of Exotic Pests (OPEP) model, with changes in the following: (1) a transparent dataset for building and training the model enabling future updates and use in other systems, (2) uncertainty simulations using values derived from an extensive natural history matrix rather than an assumed equal distribution, and (3) predictions made on the probability of multiple impact levels, allowing users to decide based on acceptable risk. The model is designed for pest risk analysis for Scolytinae in the continental United States but can be adapted to other pests or regions. We tested the model's performance by iteratively removing each species from the training set and retraining the model. The retrained models accurately predicted the removed species. To demonstrate the model's application, we predicted the impact of scolytine beetles not yet present in the continental United States, *Xylosandrus morigerus* and *Hypoborus ficus*, plus an additional hypothetical species with no known data. Our model predicts that these species are likely to have moderate impacts and unlikely to have high impacts if they were introduced.

## INTRODUCTION

Non‐indigenous forest insects have been responsible for some of the most catastrophic losses in forested systems, leading to extensive economic and environmental harm (Holmes et al., 2009; Roy et al., 2014). New introductions arise from the global trade in goods, through the movement of live plants, wood packing material, and dunnage (Lovett et al., 2016). Mitigation strategies such as early detection and rapid eradication reduce the risks of introduction (Myers et al., 2000), but to facilitate continued trade, investments into border protection, monitoring, and pathway regulation must be prioritized to species or groups that are likely to cause the worst impacts (Lovett et al., 2016). Prioritization of potential pests relies on predicting the impacts of species not yet present.

The methods currently used to prioritize potential pests rely on subjective assessment or general pest risk impact models. In the United States, expert consensus as a tool for invasive species assessment is stipulated in the Plant Protection Act of 2000, the Agricultural Bioterrorism Protection Act of 2002, and the 2008 Amendment to the Lacey Act of 1900. Similarly, in Europe, a list of foreign organisms posing unacceptable risk is based on consolidated expert opinions (Battisti et al., 2020; EU 2016/2031 Plant Health Law).

Expert opinion is guided by experience with other pests and their behaviors but does not follow any described methods or processes that can be reproduced. Expert opinion can increase the model's validity and act as an alternative when data are lacking (Kamali et al., 2017). However, even with substantial development of international standards and guidance, expert opinion is not always consistent (Baker et al., 2005). We propose an evidence‐based method that decreases subjective bias and increases repeatability. Consistent risk assessment leading to numerically ranked pest categorization methods could improve resource allocation and facilitate safe trade (Baker et al., 2005). Our model also focuses on pests before they are introduced, which allows for a more proactive approach to reduce the impact of introduced species (Roy et al., 2014).

One reason why expert opinion has been a standard approach to pest risk assessment is that data on arthropod–plant interaction before invasion are scarce (Roy et al., 2014). Many high‐impact pests were barely known in North America and Europe before they caused host tree mortality in invaded regions. These invasions were highly unexpected, described as “Black Swan events” (Ploetz et al., 2013), which are events that are unanticipated. The risk of future Black Swan events, in terms of destructive invasive species, has been discussed, and sometimes described as if there is an innate inability to predict such events (e.g., Wingfield et al., 2015). This approach suggests that the risks are not only unknown, but unknowable.

While it is important to appreciate the risks of the unanticipated events, for potential pests of forests, a pessimistic view that destructive invasive pests are unknowable has two major downsides. First, the information is often knowable, just not yet known, or even known but not yet utilized (Dong et al., 2023). Second, the amount of data being collected is rapidly accelerating, especially in geographical areas from where species are often introduced (Kenis et al., 2018), and the new data enable us to start using empirical assessment (e.g., Yemshanov et al., 2009). Not only are there more studies on insects worldwide, but there are also novel approaches designed to specifically test interactions between non‐indigenous insects and potentially vulnerable host plants even before the invasion, that is, using a sentinel garden approach (Mansfield et al., 2019). Additionally, fungi carried by non‐indigenous insects are tested for pathogenicity (Li et al., 2022), and access to non‐English literature is improving (Ernstsons et al., 2021).

Predictive models for invasive species are not new and have been described and used extensively (EPPO/OEPP, 2012; Koop, 2012; Mech et al., 2019; Panetta, 1993). There are two broad categories of these models. One is to predict the suitability of an area, usually using climate data and host usage. The second goal of a predictive model is to predict the impact of a non‐indigenous insect if it were to be introduced, with the assumption that suitable hosts, suitable climatic conditions, and introduction pathways will be interpreted concurrently by risk analysts. We developed a predictive model for the potential impact of pests if they were to be established.

Bark and ambrosia beetles (Coleoptera: Curculionidae: Scolytinae) are remarkable in their diversity—not only in terms of the number of species (>6000) but also in the diversity of behaviors and life history (Kirkendall et al., 2015). What is most remarkable is the widespread convergence of behavioral traits and strategies. This makes predictive models particularly useful for bark and ambrosia beetles, since traits are replicated among taxonomic groups, so they are less likely to be inseparable from phylogenetic relations. Bark beetles are also challenging because the impact of each taxonomic group is highly non‐uniform (Mech et al., 2019). While the great majority of species introduced in non‐native regions do not lead to reports of damage to natural and managed systems, a minority have caused dramatic tree mortality (Fraedrich et al., 2008; Karnosky, 1979). Damage in invaded regions is also related to specific biological features of the “tree‐killers,” such as the colonization of life host tissue (Hulcr et al., 2017), or association with pathogenic fungi and behaviors facilitating inoculation (Brasier, 1991). We used variable behavioral traits, especially those related to killing trees, as predictors in our model.

Despite the improvements in data availability and model bias, existing predictive models are limited by several factors. One is that there was a low number of bark and ambrosia beetle species included in the model development, training, and validation. This can lead to the overestimation of risk of the whole group when only high‐impact invaders are used for model parametrization (e.g., Schulz et al., 2021). Some models predict the probability of establishment without an explicit prediction of damage, which limits their practical utility (Lantschner et al., 2017). In addition, there are many ways to be a pest. The types of wood borer impact are diverse (e.g., prematuration feeding of twigs, branch dieback, and inoculation of pathogens), some variables have a non‐linear correlation with the response, and variables may be positively or negatively autocorrelated. This leads to predictor variables having low explanatory value. Some are only slightly more predictive than random guesses, and alone they may not be strong enough to provide statistically significant models in classical statistics. However, even models with a degree of uncertainty may be very useful for practical risk assessment, because of their capacity to eliminate low‐risk species.

Information about species worldwide is increasing, but there are still data either missing entirely or with some degree of uncertainty. Most risk assessments before 2010 followed a method of questions that have multiple choice answers, the answers are weighted, and the total score acts as a scale to classify the results (see Heikkilä, 2011 for an extensive review of these methods). However, of the 75 risk assessment models reviewed by Heikkilä (2011), few accommodated uncertainties, and in all cases, this was simply to use either a worst‐case score or a midpoint score for unknown values. More recent models rely on Monte Carlo simulations to estimate unknown or less certain variables (Caton et al., 2018).

The aim of this study is to provide a model and workflow to predict the impact of new non‐indigenous bark and ambrosia beetles introduced to the continental United States. The model accommodates many variables that are not significantly predictive on their own but provide a more accurate prediction when used together, and it accommodates uncertainty in the input variables to give a quantified risk of standardized impact levels.

## MATERIALS AND METHODS

### Terminology

We follow the recommended terms in the International Plant Protection Convention glossary (IPPC, 2024). For species that do not naturally occur in the continental United States, we refer to them as “non‐indigenous,” in invasion biology literature is synonymous with “non‐native” (Iannone et al., 2020). For species that are both non‐indigenous and are not present in the continental United States, we use the term “alien species,” which is synonymous with “introduced non‐native species.”

### Approach

Our overall approach is to use past introductions to predict future outcomes. This approach assumes that the current introduced species are a representative sample for future introduced species, in terms of the correlation of impact with potentially predictive variables. This approach is possible in the continental United States because of the large number of introduced and well‐documented Scolytinae.

We validate the model with subsampling, in which we measure the success of predicting the impact of a subsample of known introductions. There are varying amounts and quality of data about species that are not yet introduced to the United States. We accommodate this additional uncertainty using methods similar to those described by Koop et al. (2012), using probability‐derived simulations to make a distribution of predictions from which the final prediction is chosen. Additionally, to demonstrate the real‐world utility, we use the model to investigate two species not yet known from the continental United States and an additional hypothetical species with no known data.

### Selecting training species

We assembled a list of all known alien (i.e., introduced non‐indigenous) Scolytinae to the continental United States using literature (e.g., Haack, 2006), specimen databases (Atkinson, 2022, barkbeetles.info) and unpublished reports (Table 1). To eliminate indigenous species mistakenly assumed to be non‐indigenous, we only considered the Scolytinae species as alien if the species had (1) a known range outside North America with congeneric species on a different continent, and (2) high genetic diversity in a different continent and/or an abrupt appearance and spread in North America.

**TABLE 1:** A checklist of alien species of Scolytinae in the continental United States with a rating of their impact.

Scientific name	Impact score
*Ambrosiodmus lewisi* (Blandford, 1894)	1
*Ambrosiodmus minor* (Stebbing, 1909)	1
*Ambrosiodmus rubricollis* (Eichhoff, 1875)	1
*Ambrosiophilus atratus* (Eichhoff, 1875)	1
*Ambrosiophilus osumiensis* (Murayama, 1934)	1
*Anisandrus dispar* (Fabricius, 1792)	3
*Anisandrus maiche* Stark, 1936	2
*Cnestus mutilatus* (Blandford, 1894)	3
*Coccotrypes advena* Blandford, 1894	1
*Coccotrypes carpophagus* (Hornung, 1842)	1
*Coccotrypes cyperi* (Beeson, 1929)	2
*Coccotrypes dactyliperda* (Fabricius, 1801)	1
*Coccotrypes distinctus* (Motschulsky, 1866)	1
*Coccotrypes robustus* Eichhoff, 1878	1
*Coccotrypes vulgaris* (Eggers, 1923)	1
*Cryphalus itinerans* Johnson, 2020	1
*Cryphalus mangiferae* Stebbing, 1914	2
*Crypturgus pusillus* (Gyllenhal, 1813)	1
*Cyclorhipidion bodoanum* (Reitter, 1913)	1
*Cyclorhipidion nemesis* Smith & Cognato, 2022	1
*Cyclorhipidion pelliculosum* (Hagedorn, 1912)	1
*Cyclorhipidion tenuigraphum* (Schedl, 1953)	1
*Dactylotrypes longicollis* (Wollaston, 1864)	1
*Dryoxylon onoharaense* (Murayama, 1933)	1
*Ernoporus parvulus* (Eggers, 1943)	1
*Euwallacea fornicatus* (Eichhoff, 1868)	7
*Euwallacea interjectus* (Blandford, 1894)	5
*Euwallacea kuroshio* Gomez & Hulcr 2018	6
*Euwallacea perbrevis* (Schedl, 1951)	5
*Euwallacea similis* (Ferrari 1867)	1
*Euwallacea validus* (Eichhoff, 1875)	1
*Heteroborips seriatus* (Blandford, 1894)	1
*Hylastes opacus* Erichson, 1836	1
*Hylastinus obscurus* (Marsham, 1802)	4
*Hylurgops palliatus* (Gyllenhal, 1813)	1
*Hylurgus ligniperda* (F., 1787)	1
*Hypothenemus birmanus* (Eichhoff, 1878)	1
*Hypothenemus javanus* (Eggers, 1908)	1
*Orthotomicus erosus* (Wollaston, 1857)	3
*Phloeosinus armatus* Reitter, 1887	4
*Phloeotribus scarabaeoides* (Bernard, 1788)	3
*Pityogenes bidentatus* (Herbst, 1784)	2
*Premnobius cavipennis* Eichhoff, 1878	2
*Scolytus mali* (Bechstein, 1805)	3
*Scolytus multistriatus* (Marsham, 1802)	8
*Scolytus rugulosus* (Müller, 1818)	4
*Scolytus schevyrewi* Semenov, 1902	5
*Tomicus piniperda* (Linnaeus, 1758)	4
*Trypodendron domesticum* (Linnaeus, 1758)	3
*Xyleborinus artestriatus* (Eichhoff, 1878)	1
*Xyleborinus attenuatus* (Blandford, 1894)	1
*Xyleborinus octiesdentatus* (Murayama, 1931)	1
*Xyleborinus saxesenii* (Ratzeburg, 1837)	1
*Xyleborus glabratus* Eichhoff, 1877	8
*Xyleborus monographus* (Fabricius, 1792)	4
*Xyleborus pfeili* (Ratzeburg, 1837)	1
*Xylosandrus amputatus* (Blandford, 1894)	1
*Xylosandrus compactus* (Eichhoff, 1875)	4
*Xylosandrus crassiusculus* (Motschulsky, 1866)	3
*Xylosandrus germanus* (Blandford, 1894)	3

*Note*: Though some species listed here may have been “introduced,” they still may be considered quarantine pests for the United States.

### Quantifying impact

We assessed the impact of the existing alien species following the standardized protocol and the 9‐point scale described in Schulz et al. (2020) and Mech et al. (2019), whereby the score is given by the highest matching statement, as follows: (1) No damage documented in the literature; (2) minor damage (e.g., leaf/needle loss, leaf/needle discoloration, twig dieback, or fruit drop); (3) mortality of individual stressed plants; (4) weakening of an individual plant that suffers mortality from another agent; (5) mortality of individual healthy plants; (6) isolated or sporadic mortality within an affected plant population; (7) extensive or persistent mortality within a population (e.g., >25% mortality over 10 years); (8) wave of plant mortality with regional spread of the insect; and (9) functional extinction of the host plant.

All alien species of bark and ambrosia beetle species were included, even those without any documented damage, with the assumption that damage would have already become evident and recorded for these species. The impact ratings are then converted to five Boolean variables—whether the impact rating is equal to or greater than 2, 3, 4, 5, and 6. We excluded the minimum impact rating of 1 (=no reported damage) because all taxa have an impact greater than or equal to that rating. We did not make response variables for whether a species has an impact rating of equal to or greater than 7, 8, and 9, since these categories had fewer than four taxa, with little differences between categories.

### Predictor variable selection

We selected the set of predictor variables based on variables used in previous models from the primary literature (Mech et al., 2019), variables used in an Objective Prioritization of Exotic Pests (OPEP) Impact Assessment model currently used by the USDA‐APHIS (2019; https://download.ceris.purdue.edu/file/3798) which assesses many aspects of the species, including questions about the biology, behaviors related to damage, reported perceptions of damage in literature, the quantity of information available in literature, and the interactions with plant pathogens. We also suggest new, additional variables from our research on tree decline and disease caused by bark and ambrosia beetles (e.g., Hulcr & Dunn, 2011). Variables are tailored to be only true or false responses, stored as 1 or 0, respectively. For the predictive model user, these variables are framed as questions or statements. We scored these variables for all training species based on an extensive literature review, alongside unpublished data and observations by the authors.

### Model selection, implementation, and analysis

To make our predictive model, we used random decision forests (Breiman, 2001; Ho, 1995) to predict the probability of a given potential pest being in each category or higher. In short, the subjects (the species) and the variables (each questionnaire response) were randomly subsampled with replacement to make 1000 decision trees. The result is the proportion of the decision trees that give a particular outcome. This method gives robust results for otherwise noisy data and thus is much more appropriate than using decision trees alone.

**FIGURE 1:**
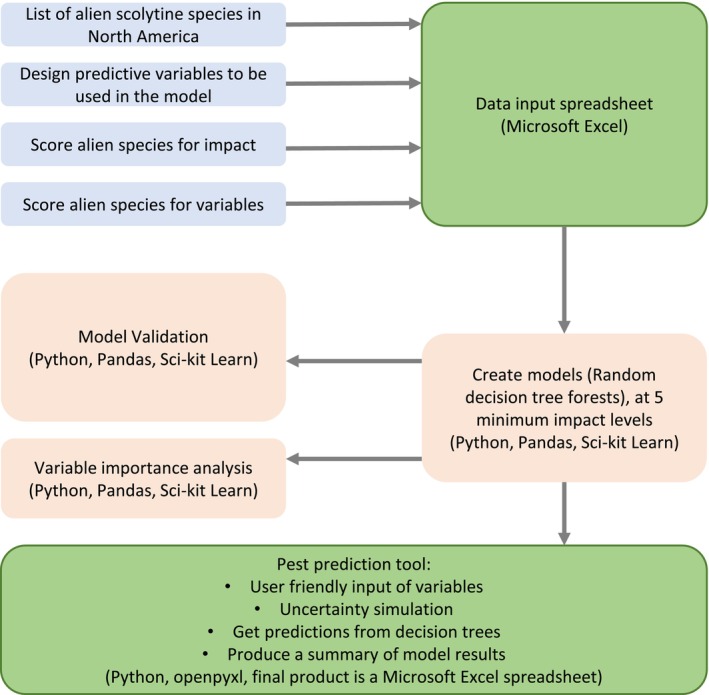
A workflow for building the predictive tool. Blue boxes represent data inputs. Cream‐colored boxes represent processes. Green boxes represent Excel spreadsheets. All scripts, datasheets, and resultant Excel spreadsheets are available in Zenodo at https://doi.org/10.5281/zenodo.15605162.

We used all the training data to make the final predictive model, but subsampled training sets were used to validate the model during development. The training dataset was entered and formatted in Microsoft Excel, with sheets designed as a relational database, with separate tables for training species, questions, and responses, each with unique identifiers. Additional sheets are used to preview the data entered. The datasheet is read into Python (V 3.9) using the Pandas Python Analysis Library. The creation of the predictive models and variable importance analysis was made using the SciKit‐Learn package (Pedregosa et al., 2011). Validation of the models was implemented using custom scripts for subsampling the datasets. We then used custom Python scripts and the package OpenPyXl (Gazoni & Clark, 2018) to create the tool to enter data for test subjects and run the uncertainty simulation to make predictions using the random forest models. The source code and final Excel sheets are available on GitHub (Johnson, 2025). A simplified workflow is presented in Figure 1.

### Assessment of variables

To assess the relative predictive power of variables, a permutation test was run whereby the values for a particular variable were randomly shuffled, and the model was recalculated with the same process; then the predictions with the test subjects were compared to see how much worse the model performs. This process was implemented in the Python SciKit Learn package (Pedregosa et al., 2011). These steps were also used on each of the impact boundaries to test the relative importance of the variables for predicting different impact levels.

### Subsampling for model validation

**FIGURE 2:**
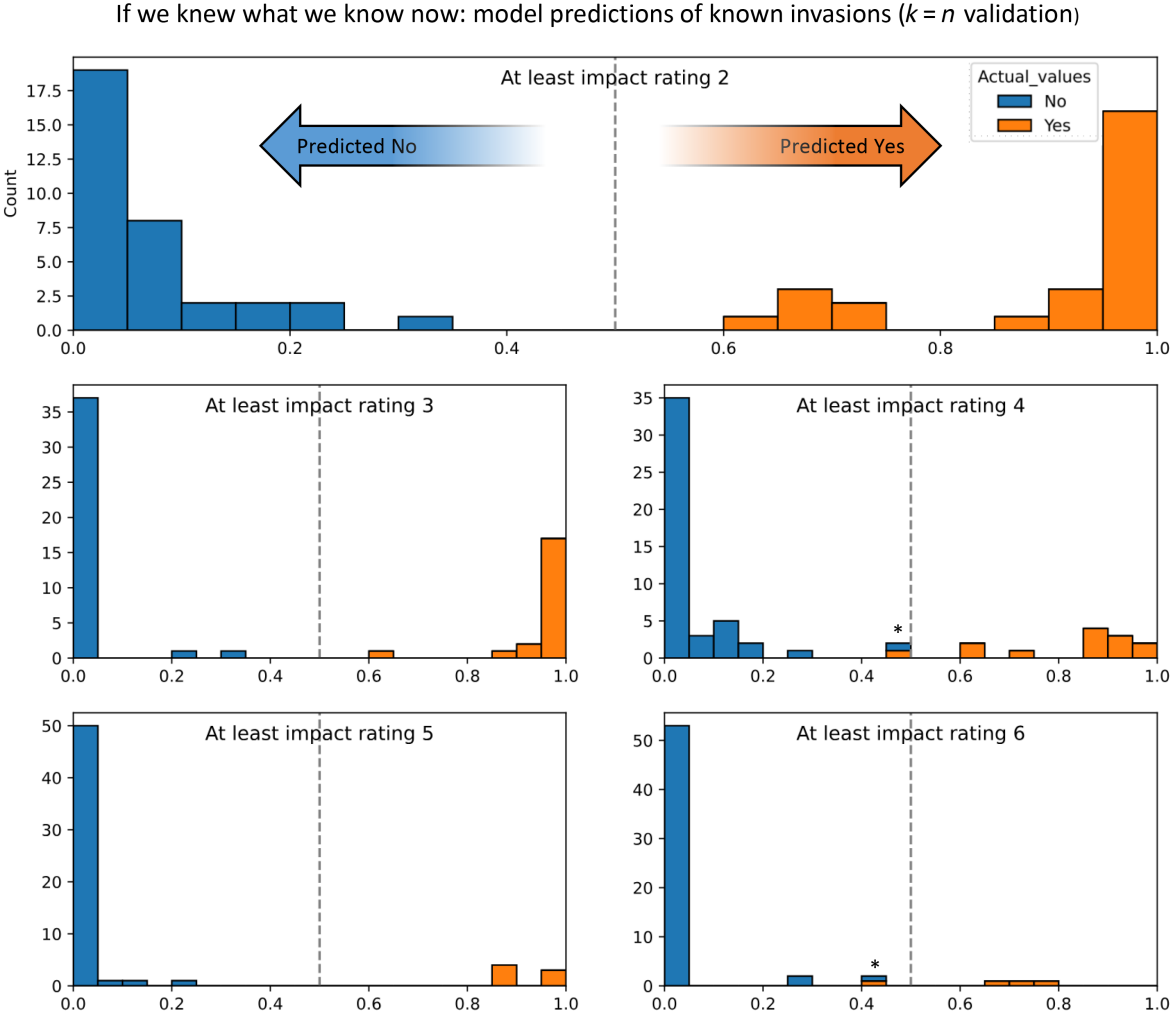
Histograms validating the model predictions of impact ratings for already introduced species. The *y*‐axis represents the number of unique species tested, with each species being assessed using a leave‐one‐out approach (i.e., the model is recalculated without that test species). The color indicates the actual impact rating assigned based on assessments, while the *x*‐axis represents the model's predicted impact rating. The top histogram is enlarged, with arrows illustrating how to interpret the figure: Species counts, their actual ratings, and how predictions align or differ from those ratings. Asterisks highlight two cases in which the model prediction, based on a 50% threshold, differs from the actual assessed impact rating.

To use the whole dataset to validate the model does not give fair validation, since the model may be overfit to predict what it has already encountered. To overcome these issues, we removed one of the training species, recalculated the model with the remaining training species, and then ran the model using the removed species as test subjects. The presented results (Figure 2) are from such validation exercises.

### Accommodating uncertainty

Uncertainty is an important part of risk analyses, yet most risk analysis models do not adequately address uncertainty (Koop, 2012). The weed risk model (Koop et al., 2012) accommodates this with Monte Carlo methods, where a random number, in combination with a certainty value, is used to generate simulated variable sets which are analyzed, giving a distribution of outcomes (Caton et al., 2018).

The risk model tool proposed here follows that, with two modifications. Firstly, all the questions have only two options (true and false). The data stored is the probability of the answer being true. This is an improved data structure compared with the weed risk model and the current arthropod OPEP model, which records the first choice, second and third choices if applicable, and the probability that the first choice is correct. Secondly, in the weed risk model, if a variable has no data, all of the available options are given an equal probability. This was not used because, for many variables, one answer is rarer than the other (Table 2). After collecting the data for our training species dataset, we were able to calculate a “default value” for completely unknown data. The “default value” is an estimated distribution that matches what is already known. The data collectors can later modify this answer based on data as it becomes available. For example, a variable used here is “The potential pest lives in the base of the trunk.” If this was being assessed for scolytines with no published biological data at all, the default value is 0.3283582, or approximately 33% likely to be true, based on the distribution of training species. If there is some ambiguous information that suggests that the beetle lives in the trunk, and the assessor estimates that this is a 90% probability of being true, then 0.9 is entered.

**TABLE 2:** Predictor variables used for the model, the mean/default value based on alien Scolytinae species present in continental United States, and the importance of each of the variables for each impact threshold, based on a permutation test.

Question	Mean/default	≥Imp 2	≥Imp 3	≥Imp 4	≥Imp 5	≥Imp 6
1. The potential pest lives on herbaceous plants.	0.033	0.00	0.00	0.00	0.00	0.00
2. The potential pest lives on long‐lived woody plants (typical generation time of the plant >5 years).	0.9	0.00	0.00	0.00	0.00	0.00
3. The potential pest lives in the base of the trunk.	0.367	0.01	0.00	0.02	0.00	0.00
4. The potential pest lives in the roots.	0.05	0.00	0.00	0.02	0.00	0.01
5. The potential pest lives in sapwood.	0.567	0.01	0.00	0.00	0.00	0.00
6. The potential pest lives in the phloem.	0.35	0.03	0.00	0.00	0.00	0.00
7. The potential pest lives in the pith.	0.133	0.02	0.00	0.00	0.00	0.00
8. The potential pest regularly lives in plant material <2 cm in diameter.	0.367	0.08	0.00	0.01	0.00	0.00
9. The potential pest regularly lives in plant material >2 cm in diameter.	0.883	0.00	0.00	0.00	0.00	0.00
10. The potential pest initiates galleries/oviposit on cut and felled trees /fallen branches.	0.783	0.03	0.00	0.02	0.00	0.00
11. The potential pest is only found initiating galleries/egg laying on still‐standing trees, stumps and snags.	0.083	0.00	0.00	0.02	**0.16**	**0.22**
12. The potential pest has been reliably recorded as reproducing on more than one host species.	0.983	0.00	0.00	0.00	0.00	0.00
13. The potential pest has been reliably recorded as reproducing on more than one host genus.	0.883	0.00	0.00	0.00	0.00	0.00
14. The potential pest has been reliably recorded as reproducing on more than one host family.	0.633	0.00	0.00	0.03	0.00	0.00
15. The potential pest has been reliably recorded as reproducing on more than three host families.	0.533	0.00	0.00	0.00	0.00	0.01
16. The potential pest reproduces on coniferous hosts (gymnosperms).	0.267	0.03	0.00	0.00	0.01	0.00
17. The potential pest reproduces on broad leaf hosts (angiosperms).	0.867	0.00	0.00	0.01	0.00	0.00
18. The potential pest lives on hosts which are congeneric with native US species.	0.85	0.00	0.00	0.00	0.00	0.00
19. The potential pest has more than one generation per year.	0.733	**0.15**	0.00	0.00	0.00	0.00
20. The potential pest attacks living plants (includes stressed plants).	0.35	**0.23**	**0.63**	0.08	0.02	0.00
21. The potential pest attacks healthy plants.	0.017	0.00	0.00	0.00	0.00	0.03
22. The literature indicates evidence of plant defense on initial attacks. (e.g., resin flow, latex, and sugar exudates).	0.267	**0.21**	0.00	0.00	0.00	0.04
23. The potential pest mates predominantly in the parental gallery (males flightless, females establish new galleries alone).	0.683	0.01	0.00	0.00	0.00	0.00
24. The potential pest maturation feeds after dispersal and before its oviposition site.	0.1	0.00	0.00	0.00	0.00	0.00
25. The potential pest causes partial loss of plants (e.g., flagging, limb dieback).	0.25	0.00	0.00	0.05	0.02	0.00
26. The potential pest damages growing tips.	0.167	0.00	0.00	0.00	0.00	0.00
27. The potential pest causes cosmetic damage to living plants.	0.083	0.00	0.00	0.08	0.00	0.05
28. The potential pest has dedicated publications about their behavior as a pest, their control, or other aspects of pest science (not taxonomy, ecology, etc.), typically indicated by their scientific or vernacular name in the title.	0.383	0.06	0.01	0.01	0.01	0.00
29. The literature describes this as a pest (minor, major, etc., secondary is ambiguous and may refer to minor pests or species which just eat dead material killed by another pest or may refer to context dependent pests. User to interpret literature and reflect uncertainty in answer).	0.417	**0.14**	**0.17**	0.01	0.00	0.00
30. The scientific literature where pest status would typically be discussed exists, and describes the pest as major, significant, or damaging.	0.267	0.00	0.01	0.07	0.00	0.01
31. The scientific literature where pest status would typically be discussed exists, and explicitly describes the insect as not a pest, or not of economic or environmental significance. This excludes cases where the justification for being described as a non‐pest is because their hosts are not directly economically important.	0.017	0.00	0.00	0.00	0.00	0.00
32. The scientific literature describes impacts (negative changes to established systems, including agriculture, forestry, horticulture and urban forestry, and the natural environment) with specific quantitative information about loss (e.g., yield loss, mortality, and growth rate).	0.017	0.00	0.00	0.00	0.00	0.02
33. The potential pest is often associated with a plant pathogen.	0.15	0.00	0.00	0.03	**0.15**	0.03
34. The potential pest is persistently/obligately associated with a plant pathogen.	0.15	0.00	0.00	0.03	**0.13**	0.03
35. Associated pathogens have been isolated from mass‐die‐off events.	0.083	0.00	0.00	0.00	0.03	0.05
36. The potential pest is often associated with a plant pathogen and the pathogen alone spreads and kill the whole host plant, that is, causes a systemic infection (usually requires experimental evidence).	0.083	0.00	0.00	0.00	0.03	0.04
37. The potential pest is often associated with a plant pathogen and the plant pathogen spreads through root grafts.	0.05	0.00	0.00	0.00	0.00	0.02
38. The potential pest is often associated with a plant pathogen and the pathogen always kills the tree, including healthy and unhealthy trees.	0.05	0.00	0.00	0.00	0.00	0.02
39. The potential pest is often associated with a plant pathogen and the pathogen kills trees in combination with environmental stressors (for which the trees would probably survive otherwise).	0.267	0.06	**0.21**	**0.30**	0.00	0.00
40. The potential pest is often associated with a plant pathogen and there are known continental‐level difference in the susceptibility of congeneric or related hosts to associated pathogens; Congeneric or related hosts in a non‐native continent show higher susceptibility to disease than hosts in the native area.	0.05	0.00	0.00	0.00	0.00	0.02
41. The potential pest is often associated with a plant pathogen and the plant pathogen alone can kill trees.	0.05	0.00	0.00	0.00	0.00	0.02
42. There are available lures that are effective at monitoring this potential pest.	0.433	0.00	0.00	0.02	0.00	0.00
43. The potential pest can be reliably identified from species already present in the United States (using a basic microscope, not molecular techniques).	0.817	0.01	0.00	0.00	0.00	0.03
44. The potential pest can be reliably identified in the field from species already present in the United States.	0.317	0.00	0.00	0.02	0.00	0.04

*Note*: The importance scores (the effect of the permutation test) >0.1 appear in boldface.

For calculating the risk of impacts, we used an approach like the weed risk model with simulations (Koop, 2012). For 1000 repeats, the variables which are input into the model are chosen based on the probabilities supplied by the assessor (or the default if left blank), by choosing a random number between 0 and 1, and assigning “true” if the random number given is less than the probability value, or “false” otherwise. Consequently, a proportional frequency of “true” would occur in the simulated repeats. By using 1000 iterations, this method provides a distribution of values for the input, which leads to a prediction distribution for each potential pest during the assessment. This simulation was implemented in Microsoft Excel, with rows of the sheet representing repeats. The predictive models were then run on all those simulated repeats, giving a distribution of predictions.

### Real‐world example test species

We demonstrated the model with three species that have not yet been introduced in the continental United States. Two real species with a reasonable likelihood of being introduced are analyzed as examples. *Xylosandrus morigerus* (Blandford, 1894) is a small ambrosia beetle that has already been introduced widely in Central America and Hawaii and could plausibly be introduced on live ornamental plants or fruits. *Hypoborus ficus* Erichson, 1836 is a minuscule beetle that can live on dry twigs of *Ficus* and is widespread in the Mediterranean. The questionnaire answers are drawn from a literature review. The procedure is identical to that intended to be used by agency risk analysts as a screening aid. The third species, while hypothetical, represents the real‐world case of a beetle for which we have no information at all and thus reflects the default rating of an unknown, as yet unresearched species. This would give a similar result to taking the average impact rating of all of the training species but would use a consistent framework that could be improved with little extra information about the species.

## RESULTS

Our research identified 60 candidate species to use to train the model, representing all alien species (i.e., introduced species) of Scolytinae in the continental United States. Their impact was rated on the 9‐point scale. Most species (57%) had the lowest impact rating of 1. Only 6 species (10%) had a rating of 5 or more. The imbalance of low‐impact and high‐impact species illustrates the essential need for the inclusion of low‐impact species when modeling the risk of impact.

We collected data organized into 42 variables on these 60 alien scolytines in North America to build a model which predicts the probability of a species belonging to each of five impact categories. The results are presented in the following ways: Predictions of the training species with single‐species removal (*k* = *n* validation, Figure 2), the robustness of the model to removing questions (Table 2), and a demonstration of the model's application for two Scolytinae species, plus hypothetical scolytines beetle with no data (Table 3). The model is used to build an Excel tool for assessing risk, available on GitHub (Johnson, 2025).

**TABLE 3:** Predictions from the five models for *Hypoborus ficus, Xylosandrus morigerus*, and a hypothetical Scolytinae with no data.

Prediction model	Mean (%)	0–10	>10–20	>20–30	>30–40	>40–50	>50–60	>60–70	>70–80	>80–90	>90–100
*Hypoborus ficus*											
≥Imp 2	92.5	0	0	0	0	0	1.1	8.1	0	0.1	90.6
≥Imp 3	86.6	9	0.2	0	0	0	0	0	0	0	90.7
≥Imp 4	12.4	44.8	42.5	6.3	5.4	0.9	0	0	0	0	0
≥Imp 5	0.4	98.4	0.2	0.2	1.1	0	0	0	0	0	0
≥Imp 6	0.4	98.2	1.7	0	0	0	0	0	0	0	0
*Xylosandrus morigerus*											
≥Imp 2	94.3	0	0	0	0	0	0.6	0.3	0	5.6	93.4
≥Imp 3	94.5	0	0.1	0	0	0.7	0.1	0	0	0	99
≥Imp 4	28.5	3.1	2.9	54.2	30.4	8.1	1.1	0.1	0	0	0
≥Imp 5	7.2	75.6	0.1	17.5	4.6	0.2	1.7	0.1	0	0.1	0
≥Imp 6	14.3	9.2	82.6	6.5	1.6	0	0	0	0	0	0
A hypothetical Scolytinae with no data											
≥Imp 2	49.8	5.1	6.6	8.8	15.5	13.4	16.2	14.3	9.7	4.2	6.1
≥Imp 3	32	44.5	2.3	0	9.4	21.6	7.4	0.3	0.4	8.3	5.7
≥Imp 4	23.7	24.8	24.5	16.4	14	12.6	6.6	0.9	0.1	0	0
≥Imp 5	10.1	56.3	24.5	6.9	9.3	1.3	1.3	0.2	0.1	0	0
≥Imp 6	4.5	83.5	14	2.1	0.2	0.1	0	0	0	0	0

*Note*: Rows represent separate models for each impact level. Numbers represent the distribution of predictions from the uncertainty simulation (e.g., for the first model [≥Imp 2, impact rating ≥2], 90.6% of 1000 simulations gave a predicted probability of 90% or more).

### Scolytinae introduced in the United States

We prepared a comprehensive checklist of alien Scolytinae in the continental United States, along with impact rating (Table 1).

### Summary of variables

We collected 42 different variables for use in the predictive model, all formatted as questions with Boolean responses. The questions are listed in Table 2, with the default value from the estimated distribution based on the training species dataset.

### Model predictions and subsampling

Using the predictor variables (Table 2) the model predicted the impact ratings listed for all alien Scolytinae species in North America (Table 1). With all the data included, our model predicts most high‐impact (all species with a predicted impact score of 5 or greater) or medium‐impact pests (all species with a predicted impact score of 3 or greater) correctly, based on a threshold probability of 50%. When validated by single species removal, the model performed very well at predicting species' impact, especially for impact ratings 3 or greater and 5 or greater, corresponding to being at least moderate impact species or high‐impact species, respectively. The model's ability to predict impact ratings 4 or greater and 6 or greater performed less effectively, with one false negative if the decision threshold is set to 50% probability. Given a complete dataset, the model is expected to perform very well at predicting the impact of a future introduction of a non‐indigenous Scolytinae.

### Assessment of predictor variables

Permutation tests showed that some questions have a strong influence on prediction accuracy. The particular questions that are most predictive differ between impact thresholds, meaning that some questions are important for identifying pests that are at least moderate impact, and others are better at identifying the higher impact pests (Table 2).

The most important variables for predicting the pests of at least medium impact (impact ≥3) are those relating to whether the organism is reported as attacking living plants (Q. 20), those which are described as a pest in literature (Q. 39) and those which interact with pathogens attach plants alongside an environmental stressor. Considering the first two variables are undoubtedly correlated, which should diminish each one's importance values, these variables are particularly useful for separating the pests predicted to have a lower impact and those predicted to have at least a moderate impact.

The most important variable for determining high‐impact pests (impact ≥5) is whether the beetle species is associated with an obligate or persistent plant pathogen (Q. 34). Even non‐obligate relationships are highly predictive (Q. 33). Another variable that is highly informative is whether the beetle obligately lives in still‐standing trees (Q. 11), suggesting this question filters out the species which are adapted to feed on fallen trees and branches.

### Tool structure and the uncertainty simulation

The final tool is an MS Excel file composed of spreadsheets to (1) input data, (2) report predictions for each impact level, and (3) report a summary of the results. The average probability among the simulations for medium‐impact and high‐impact pests (for impact levels 3 and 5, respectively) is reported. Additionally, the distribution of predictions is reported for all impact levels as frequencies in bins. The tool also includes a sheet to calculate uncertainty simulation using a pre‐calculated forest of decision trees, set as hidden. This structure conforms to the existing OPEP model framework for assessing risk.

### Real‐world examples

We demonstrated the model with three scenarios. The first two are species not yet present in the continental United States: *Xylosandrus morigerus* and *Hypoborus ficus*. The third scenario is a hypothetical scolytine species for which no information is known (Table 3). We used the available literature data for the two species to complete the data entry sheet. The confidence values were subjectively entered based on interpreting the quality of the publications. Once the models were run, the results are presented in the last sheet of the Excel document. The default method for displaying the distributions of the predictions is, for each impact level, to show the average predicted risk of being of that impact level (or greater), and the distribution in terms of the probability of falling in 10 bins (see Table 3) carried out with 1000 simulations. The mean of the simulations suggests that none of the assessed species are likely to be a high‐impact pest, but both species are likely to have a moderate impact (impact rating of 3 or greater). *Xylosandrus morigerus* had a 28.5% chance of having an impact level of 4 or more.

Assessment of the hypothetical beetle with no known information (Table 3) resulted in a multi‐modal distribution, which highlights how some variables are used disproportionately in the model. This is most evident at impact level 3, which also corresponds to the disproportionate importance of the three questions highlighted in Table 2.

## DISCUSSION

### Relentless introductions of bark and ambrosia beetles in North America

The list of alien bark beetles in North America used here is the most extensive to date. It exceeds previous lists, thanks to (1) new introductions, (2) increased trapping and monitoring programs (Rabaglia et al., 2019), and (3) recent taxonomic resolution of some groups.

The availability of this information is invaluable. Other resources aiming to make a checklist of all introduced and invasive species (https://griis.org/, McGeoch et al., 2022) list just 7 of the 60 species in the contiguous United States, highlighting the poor links between taxonomists and invasion biologists.

### Use of existing introductions to make future predictions

Our methods follow the methods of hindcasting, using existing data on the impacts of already introduced species to predict the future impacts of species not yet present. This is widely used in models with similar aims (e.g., Koop et al., 2012; Mech et al., 2019; Schulz et al., 2021).

A powerful aspect of our approach is the comprehensive treatment of introduced Scolytinae, which includes many that have had little, or no impact reported or have very little published data. This provides prior probabilities based on the existing species alone, as well as provides expected distributions of variables. Many similar models have a prerequisite of a species being described as a pest before inclusion, negating one of the primary functions: determination of whether a species is likely to become a pest at all.

The use of past introductions as the training data for future predictions includes assumptions, which need to be acknowledged when using this model. Firstly, the ecological bottleneck for establishment may have restricted many species from establishing; therefore, “filtering” the list of species used in the training dataset rather than sampling from across all non‐native Scolytinae. The effect of this assumption may lead to some systemic errors if the impact and probability of establishment are correlated. This has been partly addressed in some models such as the weed risk model for North America (Koop et al., 2012) by separating establishment potential and impact and providing two predictions to be used to assess the risk.

Interactions between introduced species are difficult to include with this technique. These interactions can be mutually inhibitory, such as if two invasive species compete. The interactions may also facilitate impact, such as between non‐native ambrosia beetles that are competent to vector each other's fungus that is pathogenic to plants (Carrillo et al., 2014).

Additional assumptions include similar establishment probabilities among species, introduction pathways staying the same as in the past, competition or compounding issues with both natives and existing alien species, or existing impacts on a particular tree host from a different alien species. While these potential caveats should be considered when interpreting the outcomes of these predictive models, there are no apparent trends or differences in the existing introductions over time, except the frequency of these introductions (Seebens et al., 2017).

### Combining weak predictors for pest categorization

Most recent work on impact predictions of non‐indigenous arthropods (e.g., Mech et al., 2019; Schulz et al., 2021) relies on models based on classical statistics, which tend to avoid using variables that are not statistically significant to explain the data. The statistically significant variables are then used to predict the impact of potential new invasives. These lead to simplified models with one or two variables used to make the best prediction balancing accuracy and over‐parameterization.

This approach loses the data which may provide some value, that is, weak predictors. All of these may be inseparable from random chance alone, but utilizing many of these weak predictors simultaneously can give better results than excluding them. Using variables with some signal but without statistical significance have already been used for predictions in cumulative score‐based methods, such as the weed risk model (Koop et al., 2012), and the existing OPEP model (https://download.ceris.purdue.edu/file/3798). In those models, a score is assigned for each response, and the cumulative score is then used as a predictor of impact, enabling the use of all variables. The advantage of this method is the easy conversion to an assessment tool for analyzing the data, which could even be calculated manually with thresholds and lookup values. However, the methods for assigning scores are not repeatable and were manually modified.

To build upon the advantages of a cumulative score system, we utilized a method of random decision tree forests, which subsamples the dataset (in terms of variables and subjects) and creates many decision trees, which can be used on a test subject, using the many separate outcomes calculated. The advantage of this method is that the construction of the model from the training dataset relies on repeatable methods with no human bias in the particular scorings. The algorithms lend themselves to be repeated many times with subsamples for validation of the methods. Additionally, as new or better data becomes available for the training dataset, the tool can be recalculated to give the best predictions available.

### Prediction of multiple impact levels

Our approach for predicting multiple, independent impact levels is novel and is advantageous over other methods. Examples of logistic regression‐based models for invasive species (e.g., Mech et al., 2019; Schulz et al., 2021) predict only a single Boolean outcome (i.e., a two‐category predictive model), such as the probability that a pest will cause a high impact (equivalent of ≥Imp 5 in our model). An alternative approach, one that has been used, is the cumulative score‐based models such as the weed risk model and the arthropod OPEP model, which use a summed score to estimate the risk, with arbitrary boundaries between three impact levels, then logistic regression to estimate the category of the assessed pest (Koop et al., 2012). Using a single scoring system and one total score assumes that the effects of traits are additive. Our analysis of each question's importance found that some variables are more important for different impact levels, suggesting that the correlations between some traits and impact levels are not linear.

We appreciate that the models which give a single probability as a result (e.g., Mech et al., 2019; Schulz et al., 2021) have the advantage of giving a simple output of the most important category: the risk of a catastrophic impact. However, a finer scale is useful for regulatory agencies because medium‐impact pests (those with an impact rating of 3 or greater but not impact rating of 5 or more) could still lead to significant economic harm, especially ones that attack highly economically significant commodities or commodities sensitive to minor impacts, such as the aesthetics of street trees in an urban environment. Creating a model which predicts to multiple, independent categories (i.e., one model, multiple categories) severely reduces sample sizes. To enable a finer scale without making categories too small, we used a novel approach using multiple predictive models to categorize to “equal or greater than” the impact for each, of the impact levels. This approach overcomes issues of small sample sizes, non‐linear relationships between variables and impacts, and is not affected by the potentially unequal spacing between the ordinal impact levels.

### Uncertainty simulation

Accommodating uncertainty is a critical, yet often missing part of risk assessment for potential pests. We addressed it following the methods described by Caton et al. (2018), except with a simplified data structure (only two options) and the use of prior distributions of character states when the data are unknown. These changes allow for much more elegant data storage (single value responses for every question) and give a much more realistic prediction for species with little data.

One disadvantage of using the method of simulation via randomly picking each variable is that many of the weak variables are correlated, which may lead to a broader probability distribution than if all the simulated repeats contained a more plausible combination of correlated variables. Methods are available that will impute these data based on what is in the training dataset (Van Buuren & Groothuis‐Oudshoorn, 2011), although we did not trial this here. An alternative approach, facilitated by an explicit “live” training dataset, is to re‐develop the model only with the characters that are known.

### Accuracy of modeling past introductions with known effects

The general approach can also be evaluated based on what we know about recent introductions. An example would be *Xyleborus glabratus*, the redbay ambrosia beetle, which was introduced to the United States around 2002 and triggered a massive die‐off of lauraceous trees due to its phytopathogenic fungal symbiont. At the time of introduction, very little was known about it. Given the lack of information, a hypothetical assessment would have given results similar to the results of an “hypothetical unknown Scolytinae,” except a slightly lower probability because some questions pertain to the lack of published information.

Therefore, this model would not have been effective at accurately predicting the risk at the time. Since then, however, additional observations in Asia have indicated traits which would increase that risk, such as attacking living parts of trees (Hulcr et al., 2017), exemplifying that the knowledge to assess *X. glabratus* was missing previously, but not the “unknowable” black swan.

### Testing the model with real examples

We demonstrate the model using two species, *H. ficus* and *X. morigerus*. The species were chosen because of their wide distribution, perceived high likelihood of being introduced to the United States, and the availability of sufficient information for scoring. Both were found to have a high probability of being a moderate pest (impact ≥3). As part of a process for assessing potential pests, these species may be assessed further regarding the introduction pathways and size of the commodity under threat, as well as decisions of acceptable levels of risk based on the distribution of predictions. These two species give an example of how the model can be used and interpreted.

The inclusion of a completely unknown beetle demonstrates a baseline risk before evaluation and demonstrates the range of outcomes based on the simulation. While the mean result should simply represent the mean of the training species, evaluating this in the same framework gives the most extreme version of an unknown species.

## CONCLUSION: DEPLOYMENT OF THE MODEL AS A TOOL FOR DECISION‐MAKING

The aim of the model is to provide risk analysts with a quantified risk at multiple impact levels for a bark or ambrosia beetle not yet present in North America based on biological traits, behaviors, and reports about the species elsewhere. Risk analysts can use this estimated risk in conjunction with pathway risks, establishment potential, and the value of the commodities at risk to make regulatory decisions to facilitate international trade, such as targeted restrictions, treatment requirements, and monitoring schemes.

## AUTHOR CONTRIBUTIONS

Andrew J. Johnson co‐conceived the project, developed the model approach, wrote the scripts, researched the training dataset, and co‐wrote the manuscript. Jiri Hulcr and David Bednar co‐conceived the project, reviewed and improved the approach, methods, dataset and results, and co‐wrote the manuscript.

## CONFLICT OF INTEREST STATEMENT

The authors declare no conflicts of interest.

## Data Availability

Data and code (Johnson, 2025) are available in Zenodo at https://doi.org/10.5281/zenodo.15605162.
